# Dietary Therapy in Prevention of Cardiovascular Disease (CVD)—Tradition or Modernity? A Review of the Latest Approaches to Nutrition in CVD

**DOI:** 10.3390/nu14132649

**Published:** 2022-06-27

**Authors:** Elżbieta Szczepańska, Agnieszka Białek-Dratwa, Barbara Janota, Oskar Kowalski

**Affiliations:** 1 Department of Human Nutrition, Department of Dietetics, Faculty of Health Sciences in Bytom, Medical University of Silesia in Katowice, ul. Jordana 19, 41-808 Zabrze, Poland; eszczepanska@sum.edu.pl (E.S.); okowalski@sum.edu.pl (O.K.); 2 Doctoral School of the Medical University of Silesia in Katowice, Faculty of Health Sciences in Bytom, Medical University of Silesia in Katowice, ul. Piekarska 18, 41-902 Bytom, Poland; d201083@365.sum.edu.pl; 3 Department of Cardiology, Congenital Heart Diseases and Electrotherapy, Silesian Center for Heart Diseases, ul. Marii Curie-Skłodowskiej 9, 41-800 Zabrze, Poland

**Keywords:** cardiovascular disease, CVD, diet, prevention, diet therapy, cardioprotective food

## Abstract

The development of cardiovascular diseases is undoubtedly influenced by improper dietary behavior. The most common mistakes include irregularity of meal consumption, high dietary atherogenicity: snacking on sweets between meals, low supply of dietary fiber, unsaturated fatty acids, legume seeds, and high supply of meat and meat products. Among many food components, some are characterized by a specific cardioprotective effect, which means that their supply of food may prevent the occurrence of cardiovascular disease or improve the health of the sick. Coenzyme Q10 (CoQ10) is one of the ingredients showing cardioprotective effects on the heart and blood vessels. Antioxidant and lipid profile-enhancing effects are also attributed to sitosterol which is one of the plant-derived sterols. A very important argument indicating the necessity of a varied diet rich in a variety of plant products is the beneficial effect of polyphenols, which are most abundant in multicolored vegetables and fruits. Numerous studies show their effectiveness in lowering blood pressure, improving lipid profile, and regeneration of vascular endothelium. The collected publications from the field of lifestyle medicine can be a source of knowledge for dieticians, physicians, and people associated with physical culture and human mental health to prevent the development of cardiovascular diseases and reduce the risk of death from this cause.

## 1. Introduction

Cardiovascular diseases (CVD) belong to the group of civilization diseases, the development of which, apart from unmodifiable risk factors, is significantly influenced by a modifiable lifestyle [1]. Non-modifiable cardiovascular risk factors include gender, age, ethnicity, and family history of cardiovascular disease [2,3]. Modifiable risk factors are mainly body weight, dietary pattern, physical activity, smoking, alcohol consumption, and exposure to stress. Factors that can be partially considered modifiable are hypertension, hyperlipidemia, diabetes, and obesity [2,3]. These have real, both direct and indirect, effects on the development of cardiovascular disease. Adherence to correct lifestyle behaviors, including proper diet, has a preventive and convalescent effect [3,4].

The most common cardiovascular disorders include dyslipidemia, hypertension, atherosclerosis, and ischemic heart disease. In Europe, ischemic heart disease accounts for 38% of all reported deaths, whereas in Poland for 23% [1]. Cardiovascular diseases are the main cause of death in the country [1,5]. In Poland, the European SCORE scale, validated for the Polish population (POL-SCORE), is used to assess the total 10-year risk of death from cardiovascular causes. This tool evaluates the risk of death by taking into account non-modifiable factors such as gender and age, as well as lifestyle factors including total cholesterol, systolic blood pressure, and smoking [6]. This fact prompts consideration of the relationship between modifiable lifestyle factors and cardiovascular disease.

The incidence of heart disease rises sharply in postmenopausal women; it is nevertheless lower in women than in men in every age group, and this difference is reflected in the use of the ACC/AHA Risk Calculator to estimate future nonfatal myocardial infarction and stroke for a hypothetical woman and man with the same risk profiles [7]. A study of Center for Disease Control data (2015) showed that among women aged 35 in the mid-1970s, cancer mortality exceeded CVD, while the reverse is true for those aged 75 and older [8]. Some CVDs are more common in women than men, including stress cardiomyopathy (Takotsubo syndrome), coronary artery dissection, and connective tissue diseases [7]. According to the CDC report in the U.S., heart disease death rates were similar for black and white races at the beginning of the study period, i.e., 1968. However, the overall decline in heart disease death rates, measured as AAPC, was slower among black (AAPC = 2.2% per year) than white (AAPC = 2.4% per year) individuals. As a result, after 1975, heart disease mortality was consistently higher among blacks than whites. Since 2010, declines in heart disease mortality among black and white individuals have leveled off [8].

Considering the Polish epidemiological data on the prevalence of cardiovascular disease, the distinction by race is not considered because of the structure of the Polish population. Approximately 32% of the Polish adult population, or 10.5 million people, have hypertension, and only 26% of these are adequately controlled [9] In a study by Miazgowski T et al., the prevalence of HHD increased sharply with age. A similar age-dependent increase was also observed for HHD and DALY-related deaths. Between 1990 and 2016, age-standardized DALY rates associated with HHD in Poland declined but remained higher than DALYs for Western Europe, while DALYs for all age groups remained relatively stable [10].

Considering the epidemiological data on CVD prevalence, dietary recommendations and nutritional prevention of CVD should be introduced from the earliest years of life [11]. Taking into account the current lifestyle and nutrition of children and adolescents, as well as the increasing number of overweight and obese children, we emphasize that CVD prevention issues (lifestyle including diet, physical activity) should be addressed from the early years of life—the sooner, the better. Eating habits are formed from the first years. At the same time, the period of the first 1000 days of a child’s life, including the period of fetal life, influences: hypertension, early vascular ageing, and premature morbidity and mortality due to CVD [7,11].

## 2. Methods

Due to the existence of many interdependencies between lifestyle and cardiovascular diseases, as well as ongoing research in this field, there is a need for a comprehensive presentation of the latest available literature that can serve as a source of knowledge for people involved in the broadly defined prevention of diseases, their treatment, as well as diet prophylaxis and diet therapy. The 2017 American Society for Preventive Cardiology guidelines highlighted a greater role for dietitians in the health care team concerning the number of people with hypertension [7], therefore, we undertook this review to highlight modern nutrition in CVD considering super foods containing polyphenols and other substances that positively influence CVD prevention.

This review aims to discuss the recent literature in the field of prevention, diet therapy, and cardiovascular diseases, with special emphasis on ischemic heart disease. In the reference analysis, we examined traditional approaches to CVD dietary therapy and considered modern dietary models as well as functional foods as a form of CVD prevention. For this purpose, we reviewed electronic databases including PubMed from the last 5 years, including articles in English and Polish. The following keywords were used to search for articles: cardiovascular disease + diet (PubMed—16,712 publications), cardiovascular disease + dietary recommendations (PubMed—1413 publications), cardiovascular disease + prevention (PubMed—95,669 publications), cardiovascular disease + diet therapy (PubMed—5280 publications), cardiovascular disease + Mediterranean diet (PubMed—985 publications), cardiovascular disease + MIND diet (PubMed—46 publications), cardiovascular disease + DASH diet (PubMed—341 publications), cardiovascular disease + low-carb diet (PubMed—14 publications).

The literature review was based on 98 English- and Polish-language articles published between 2017 and 2022. Cross-sectional studies, meta-analyses, and both review publications and experimental studies were cited. The last search was performed on 31 May 2022.

## 3. Influence of Dietary Components on the Occurrence of Cardiovascular Diseases

The development of cardiovascular disease is undoubtedly influenced by incorrect dietary behavior [12]. Studies evaluating the diet of people with cardiovascular diseases indicate that dietary mistakes are made [13]. The most frequent errors include irregularity of meal consumption, high dietary atherogenicity: snacking on sweets between meals, low supply of dietary fiber, unsaturated fatty acids, legume seeds, and high supply of meat and meat products [14,15,16]. These studies often emphasize the importance of the role of dietitians and indicate the need for nutrition education in the prevention of cardiovascular disease [17]. The diet should, above all, be properly balanced and varied, thus providing many valuable pro-health components, including cardioprotective ones [18].

### 3.1. Cardioprotective Food Ingredients

Of the many food components, some have a specific cardioprotective effect, which means that providing them with food can prevent the onset of cardiovascular disease or improve the health of those who are ill.

Among the ingredients showing protective effects on the heart and blood vessels is coenzyme Q10 (CoQ10). Its oxidized form, ubiquinone, takes an active part in energy production in mitochondria by transporting electrons between protein complexes in the respiratory chain. In turn, the reduced form, ubiquinol, by taking up oxygen, reduces stress [19]. These properties of CoQ10 make it an essential component for the function of all cells, especially cardiomyocytes. Due to their high demand for ATP production, they contain a higher number of mitochondria compared to the others, thus showing a higher demand for CoQ10. The antioxidant properties of this coenzyme improve endothelial function and have a beneficial effect on the lipid profile [20]. Apart from the coenzyme of endogenous origin, it is possible to supply the component with food. It is worth noting that with age, taking some hypolipemic drugs, and the occurrence of cardiovascular diseases, the demand for CoQ10 increases. Products providing this component include fatty fish, soy products, nuts, and green vegetables such as spinach [21].

Essential unsaturated fatty acids show activity in support of the cardiovascular system, and omega-3 fatty acids belonging to this group are distinguished by specific cardioprotective properties. Thanks to lowering the concentration of pro-inflammatory interleukins, they contribute to improving the functioning of the endothelium of blood vessels, reducing the degree of platelet aggregation and clot formation, as well as lowering the concentration of triglycerides. They have also been shown to have a hypotensive effect, and based on many meta-analyses, they have been identified as a component reducing the risk of selected cardiovascular diseases [22,23]. Omega-3 fatty acids are abundant in marine fish (especially fatty fish such as salmon), as well as seaweeds, chia seeds, and linseed [23]. Due to the content of polyunsaturated fatty acids in marine fish, it is usually recommended to eat them or seafood twice a week [22,23].

Antioxidant and lipid profile-enhancing effects are also attributed to sitosterol, which is one of the plant-derived sterols. Plant phytosterols are abundant in oils, vegetables, fruits, (especially sprouts), nuts, and pulses [24,25].

Interventional, experimental, and in vitro studies have shown that vitamin E provides cardiovascular protection by acting at several stages of the thrombotic process [26]. A component that reduces the risk of cardiovascular disease is fat-soluble vitamin E. Nowadays, its effects are not only seen in the anti-inflammatory field, and this vitamin is seen as a bioactive molecule [27,28]. Vitamin E has been shown to counteract hyperlipidemia, atherosclerosis, and endothelial dysfunction by reducing foam cell infiltration and decreasing lipid peroxidation [27]. In addition, vitamin E reduces platelet aggregation, preventing thrombosis [27]. According to Viola F. et al., intervention studies with vitamin E should not be considered negative, but conversely, further studies with detailed methodology should be conducted to assess the validity of vitamin E supplementation in patients at risk of CVD or with CVD [26]. Food components rich in this vitamin include, in particular, wheat germ, vegetable oils, some vegetables such as broccoli and spinach, as well as nuts (mainly peanuts and hazelnuts), almonds, and products made from processing the above, e.g., peanut butter [27].

An extremely important argument indicating the necessity of a varied diet rich in various plant products is the beneficial effect of polyphenols, of which multicolored vegetables and fruit contain the largest amount. The group of polyphenols protecting the heart and blood vessels includes anthocyanins, flavonols, and flavonoids [29]. Numerous studies demonstrate their effectiveness in lowering arterial pressure, improving the lipid profile, and regenerating the endothelium of blood vessels [29,30,31].

A summary of cardioprotective food ingredients with a description of the demonstrated effects and availability in food is presented in Table 1.

Improved cardiovascular health can be improved by bioactive compounds found in foods and beverages. Such foods that contain polyphenols include berries, nuts, tea, coffee, red wine, and chocolate [27].

In particular, the consumption of fruits rich in anthocyanins and procyanidins, such as berries, has been shown to improve endothelial function through their effects on reducing oxidative stress and inflammation [27,28]. A meta-analysis involving more than 3 million people showed a significant reduction in CVD mortality with consumption of 2.5 cups of coffee per day compared with no coffee consumption [27,29].

Tea polyphenols, known as catechins, are the main bioactive substances responsible for the beneficial effects of tea on CVD risk. The beneficial effects of tea consumption on cardiovascular events, CVD mortality, and incident stroke were consistent between high-quality systematic reviews and meta-analyses [27,30].

A review of observational studies found that red wine consumption correlated with increased HDL-c levels but did not appear to alter other parameters such as triglyceride, LDL-c, and CRP levels [27,31].

Soy consumption may improve endothelial function in adults with cardiometabolic risk by regulating inflammatory biomarkers; isoflavones in soy enhance endogenous antioxidant and anti-inflammatory systems [27,28].

Increasing evidence has prompted the inclusion of nuts in many healthy eating guidelines. The U.S. Food and Drug Administration (FDA) issued a health statement on nuts in 2003, which stated the potential of nut consumption to reduce the risk of heart disease [35]. Nuts are a rich source of phytochemicals (proanthocyanidins, flavonoids, and phenolic acids) and fat-soluble bioactive and PUFA, tocols, carotenoids, phytosterols, and sphingolipids [27].

Considering the diverse bioactive substances contained in the above foods, diet will continue to play an important role in the prevention and complementary treatment of CVD.

### 3.2. Carbohydrates

Excessive consumption of simple carbohydrates (sugar, sweets, high glycemic index products) increases the risk of developing diabetes and cardiovascular disease, and contributes to the development of overweight and obesity and all their consequences. Maintaining cardiometabolic stability including, but not limited to, normal blood glucose levels carries benefits that reduce the likelihood of metabolic syndrome [36]. The World Health Organization recommends limiting the intake of simple carbohydrates to 10% of total caloric needs. However, it is suggested to consume complex carbohydrates and products with a low glycemic index, and provide dietary fiber [18,32,37,38].

### 3.3. Dietary Fiber

Dietary fiber consists of vegetable substances, soluble and insoluble, whose digestion is made possible by probiotic bacteria present in the gastrointestinal tract [39]. The products whose consumption provides this component are mainly vegetables, fruits, whole-grain cereal products, and nuts [40]. The daily fiber intake of 27–40 g recommended by the WHO carries significant health benefits, also for the cardiovascular system [18,38,39]. It has been demonstrated that a proper dietary fiber intake prevents hypertension, reduces the accumulation of visceral fat, improves insulin sensitivity, and has a beneficial effect on LDL cholesterol levels due to the reduction of cholesterol absorption from the gastrointestinal tract [39]. On the other hand, insufficient fiber intake correlates with the occurrence of vascular diseases, including coronary heart disease [40]. In a study by Zhang et al., they observed that higher dietary fiber density and higher total dietary fiber intake were associated with lower long-term CVD risk. Higher dietary fiber density was most associated with a lower risk of atherosclerotic cardiovascular disease in people aged 20–39 and 40–59 years. Young people may benefit more from high dietary fiber intake for protection against CVD, i.e., dietary fiber intake of 14 g/1000 kcal had a better protective effect in this age group [41].

An important aspect of the action of fiber is the slow digestion in the gastrointestinal tract, causing delayed gastric emptying with simultaneous prolongation of the mechanical feeling of satiety, thus reducing the amount of food consumed and preventing excessive body weight [40]. At the same time, fiber promotes improved intestinal motility [40]. Moreover, in the gastrointestinal tract, fiber is digested by probiotic bacteria for which it is a source of energy, favorably influencing the development of the microbiota and preventing inflammation-inducing dysbiosis [42]. The well-developed bacterial flora of the gastrointestinal tract itself can influence favorable gene expression, improve fatty acid metabolism, and reduce leptin levels [43].

Due to the beneficial effects of fiber and, at the same time, its insufficient intake, it is added to food products as a functional fiber to provide health benefits [40].

### 3.4. Fats

Dietary fat intake carries much controversy about the prevention and occurrence of cardiovascular disease. However, researchers agree that the risk of cardiovascular disease increases in situations of insufficient supply of polyunsaturated fatty acids and supply of saturated fatty acids with trans-configuration [44,45].

Essential polyunsaturated fatty acids should be supplied with the daily diet because of their effect on restoring metabolic homeostasis. These acids reduce oxidative stress and show anti-inflammatory effects against vascular damage [21]. Omega-3 polyunsaturated acids are used to decrease inflammatory markers in overweight individuals, metabolic disorders, and those leading inactive lifestyles, such as the elderly [21]. Natural sources of omega-3 include mainly fatty marine fish, chia, flaxseed, and nuts. Therefore, it is recommended to consume oily sea fish at least twice a week to provide the optimal amount of essential fats [23].

Trans-fatty acids are most often supplied with the consumption of highly processed industrial food. Their supply, in addition to increasing the risk of cardiovascular disease (such as hypercholesterolemia and increased diastolic blood pressure), contributes to an increased risk of death resulting from cardiovascular disease [46,47]. These fats are being phased out of industrial production; their intake should cover a maximum of 1% of energy requirements [18,32,47].

## 4. Selected Nutritional Models in the Prevention and Treatment of Cardiovascular Disease

Consideration of the most beneficial nutritional model for the prevention and protection of cardiovascular disease has accompanied scientists for decades. It has been established that the diet, through an appropriate kilocalorie supply, should ensure normal body weight, have an antioxidant effect, and be best suited to individual patient needs [17]. Dietary models have been identified, which by their assumptions are part of the principles of prevention and protection of cardiovascular disease. These include the Mediterranean, DASH, vegetarian, Nordic, low-carbohydrate, and MIND diets.

### 4.1. Mediterranean Diet

The Mediterranean diet, which belongs to the group of alternative diets, owes its name to the imitation of the diet of societies living in the Mediterranean basin. In this dietary model, there is a predominance of high-fiber products, whole grain cereals, vegetables, fruits, nuts, seeds such as pumpkin, olive oil, and oily sea fish (in the vegetarian variant—pescovegetarian or flexitarian diet) products that provide ingredients that have a beneficial effect on the functioning of the heart and blood vessels. At the same time, the intake of saturated fatty acids, trans-configured fatty acids, and animal meat, especially red meat and meat products, is limited [48]. As it provides cardioprotective components, the Mediterranean diet reduces the risk of heart failure and the risk of death, both primary and secondary, from cardiovascular disease [49,50]. Nuts, particularly almonds, walnuts, hazelnuts, and pine nuts, are very good sources of omega-6 and omega-3 fatty acids and plant sterols, which may contribute to lowering LDL cholesterol and the risk of coronary heart disease. Prospective studies have shown that consumption of 5 portions of nuts per week is associated with a 40% to 60% reduction in the incidence of coronary heart disease [51]. In addition, low glycemic index foods rich in dietary fiber have been shown to reduce insulin production and increase levels of short-chain fatty acids produced by fiber fermentation, which have been shown to inhibit cholesterol synthesis [51,52,53]. A high intake of phytosterols from nuts, seeds, whole grain products, vegetables, and fruits may also play a significant role in lowering plasma cholesterol levels by competing with intestinal cholesterol absorption [51,52,53]. Certain products of the Mediterranean diet, such as nuts, vegetables, fruits, oil, and wine, are particularly rich in antioxidants and anti-inflammatory components. Its consumption is widely associated with the improvement of several inflammatory and oxidative biomarkers. In this sense, some in vitro studies have shown that polyphenols, such as resveratrol or hydroxytyrosol, exert antioxidant and anti-inflammatory effects by capturing and neutralizing free oxygen and nitrogen species, inhibiting platelet aggregation, reducing vascular inflammation and apoptotic processes, or protecting LDL from peroxynitrite-mediated oxidation [53,54].

A growing body of evidence suggests that the five most important adaptations induced by the Mediterranean diet are: lipid-lowering effects; protection against oxidative stress, inflammation, and platelet aggregation; modification of involved hormones and growth factors in cancer pathogenesis; inhibition of nutrient-sensing pathways by specific amino acid restriction, and production of metabolites mediated by the intestinal microflora affecting metabolic health [51,55,56]. The Mediterranean diet, due to its abundance of legumes, nuts, fruits, and vegetables may modulate the gut microflora and influence the production of its metabolites, affecting CVD risk [53,55]. It is considered that the beneficial effects of the Mediterranean diet can only be explained by the synergy between all the nutrients present, which may attenuate or exacerbate the harmful or beneficial effects induced by a single nutrient [53].

### 4.2. The DASH Diet

The DASH (Dietary Approaches to Stop Hypertension) diet was created directly to counteract hypertension and reduce the risk of its complications. Its main premise is to limit the daily intake of salt, which when supplied in excess leads to an increase in the amount of water circulating in the vessels, causing an increase in blood pressure. The DASH dietary model emphasizes the consumption of fruits and vegetables, nuts, dairy products with limited fat, and plant-based proteins, mainly from legumes [49]. It has been shown that low amounts of sodium in the DASH diet mitigated the hypotensive effects of potassium, or conversely, high potassium or calcium in the DASH diet mitigated the effects of low amounts of sodium [57]. The diet results in a reduction in cardiovascular risk. In addition to lowering blood pressure, blood levels of LDL fraction cholesterol are also lowered and the risk of developing metabolic diseases, including type 2 diabetes, is reduced [49]. A lower rate of heart failure development has been observed among DASH diet followers compared to non-diet followers [50]. The high content of vitamin C, calcium, and magnesium in the DASH diet, which comes from fruits and vegetables, may contribute to the reduction of inflammation by reducing oxidative stress, and reducing NADPH oxidase activity restoring antioxidant enzymes [58]. In a study by Juraschek et al., it was shown that compared to the typical American diet, a diet rich in fruits and vegetables reduces subclinical cardiac damage and congestion, and also reduces NT-proBNP, a marker of cardiac burden [59].

### 4.3. The MIND Diet

The combination of the Mediterranean diet with the DASH diet has led to the emergence of a nutritional model, the MIND diet (Mediterranean–DASH Intervention for the neurodegenerative delay). The above anti-inflammatory dietary model is intended to counteract neurodegenerative processes, but at the same time, it provides the diet food products showing cardioprotective effects [60,61]. The MIND dietary model is mainly based on the supply of green vegetables, berries, nuts, oil, wine and fish, low-fat meat, and dairy [48,62,63]. For this reason, the diet is rich in vitamin E, flavonoids, and folate which protect the cardiovascular system through anti-inflammatory effects and prevent abnormal homocysteine metabolism [48,64]. The use of the MIND diet among post-stroke patients seems to be particularly beneficial, as its protective effect against post-stroke cognitive decline has been proven [65]. Additionally, in a study by Golzarand et al., it was shown that higher adherence to the MIND diet was associated with a lower risk of cardiovascular events. Each increase in the intake of products such as whole grains, green leafy vegetables, and beans reduced CVD risk by 60%, 45%, and 65%, respectively [66].

### 4.4. Vegetarian Diets

There are several types of vegetarian diets, but all of them are characterized by the reduction or exclusion of animal meat consumption [67]. Following a balanced plant-based diet has several benefits [68]. It has been observed that the population of countries whose usual dietary model excludes the consumption of meat is less likely to develop cardiovascular diseases [67]. Moreover, a plant-based diet significantly reduces the risk of death from cardiovascular causes [68]. Plant-based diets, unlike diets based on animal meat consumption, provide significant amounts of cardioprotective fiber, beta-glucans, and plant phytosterols that limit the absorption of lipids from the gastrointestinal tract. As a result of their protective effect, meat-free diets reduce the risk of vascular disease, hemorrhagic stroke, and ischemic stroke [69]. Plant foods rich in polyphenols and flavonoids such as fruits and vegetables have shown beneficial effects on endothelial and platelet function, and have been observed to play a protective role in the development of CVD [70]. A meta-analysis of randomized clinical trials and observational studies showed clear benefits of vegetarian diets for lowering blood pressure [70]. In a study by Satija and Hu, vegetarian diets were observed to significantly reduce blood levels of total LDL, HDL, and non-HDL cholesterol [71]. Additionally, vegetarians tend to have lower BMI values compared to non-vegetarians [70]. The American Dietetic Association recommends a varied plant-based diet for those wishing to improve their health [68].

### 4.5. The Nordic Diet

A dietary model similar to the Mediterranean and vegetarian diets is the Nordic diet, which was developed based on the dietary behavior of people living in the Scandinavian countries. This diet limits the consumption of red meat and products, providing saturated fat while increasing the supply of Scandinavian fruit and vegetables (mainly berries), vegetable fats (rapeseed oil), and fish (salmon, mackerel, herring) [72,73]. Following a Nordic diet has been shown to support the cardiovascular system by lowering the inflammatory marker C-reactive protein (CRP) [74]. An effect of the Nordic diet on lowering blood pressure and blood cholesterol levels, including LDL fraction cholesterol, has also been observed [67,75]. The Nordic dietary model may furthermore sensitize cells to insulin [75].

### 4.6. Low-Carbohydrate Diets

The reduction of carbohydrate provision in the daily dietary pattern in favor of an increased fat supply has been controversial for years. It is now considered that the amount of carbohydrate intake should be individualized [76]. Several types of diets restrict carbohydrate intake, including low-carbohydrate and high-fat diets and radical ketogenic diets [76]. Available scientific publications indicate beneficial health effects of low-carbohydrate diets, which are also observed in the cardiovascular system. It has been proven that these diets have a beneficial effect on body weight, leading to its reduction. Moreover, limiting carbohydrate intake lowers C-reactive protein and triglyceride concentrations, thus preventing vascular endothelial dysfunction and metabolic complications [76]. A positive effect of the ketogenic diet on blood pressure has also been reported; moreover, no increase in LDL fraction cholesterol was observed [77,78]. Low-carbohydrate diets contribute to lower cardiovascular risk, and recent publications tend to suggest the use of ketogenic diets in the treatment of hypertension and heart failure, although noting the controversial nature and the need for further studies of the dietary model [78,79].

A summary of the health benefits of the dietary models is shown in Table 2. In summary, the main international dietary guidelines for the treatment of hypertension are based on the DASH diet and the Mediterranean diet. Both the guidelines for the prevention, detection, evaluation, and management of high blood pressure in adults issued by the American College of Cardiology/American Heart Association (2017) recommend the heart-healthy DASH diet with a high intake of vegetables, whole grains, some low-fat dairy products, and a low intake of red meat, sugar, and trans hydrogenated fats [80,81].

Guidelines for the management of hypertension issued by the European Society of Cardiology/European Society of Hypertension (2018) support the suggestion of a low-salt diet similar to the Mediterranean diet—a healthy balanced diet that includes vegetables, legumes, fresh fruit, low-fat dairy products, whole grains, fish, and unsaturated fatty acids (especially olive oil); low intake of red meat and saturated fatty acids [80,82,83].

The International Society of Hypertension issued the Global Guidelines for the Practice of Hypertension, which recommend a diet similar to DASH, also highlighting the possible effect on lowering blood pressure by vegetable sources of nitrates such as beets and leafy vegetables. Additionally included are foods rich in calcium, magnesium, and potassium such as avocados, nuts, seeds, legumes, and tofu, also having some antihypertensive properties [80,83,84].

## 5. Influence of Non-Nutritional Factors on the Incidence of Cardiovascular Disease

In addition to dietary components and dietary behavior, other lifestyle elements such as lifestyle, amount of physical activity, smoking, alcohol consumption, and stress management influence the prevention and development of cardiovascular disease [85,86,87,88].

### 5.1. Physical Activity

An adequate number of hours of physical activity per day contributes to an improved quality of life. In the most recent guidelines, the World Health Organization recommends that adults should undertake moderate physical activity for 150–300 min per week, or play sports intensively for 75–150 min [89].

Physical activity results in lower blood pressure and reduces the progression of cardiovascular disease [89,90]. Studies show that among people with diagnosed cardiovascular disease who undertake physical activity, the risk of death is reduced compared to those who lead a sedentary lifestyle [86,91]. It has been proven that among people with increased physical activity, the likelihood of developing coronary heart disease and heart failure is also reduced [86].

### 5.2. Smoking

Smoking is a risk factor for the development of cardiovascular disease and is a criterion in the assessment of the risk of death from this cause according to the SCORE scale. WHO data indicate that the number of smokers worldwide has been steadily decreasing over the last few decades, particularly due to women giving up smoking. Unfortunately, e-cigarettes, modern products that encourage smoking, especially among young people, are currently popular on the market. However, as studies have shown, e-cigarettes are not a healthier alternative because, even though they do not provide tar, their use contributes to increased blood pressure, vascular endothelial damage, and increased inflammation [87].

Tobacco smoking increases blood pressure, leading to the development of hypertension [92]. Vascular dysfunction increased levels of inflammatory markers, and progressive atherosclerosis has been observed among smokers. A higher incidence of thromboembolic disease and heart failure has also been observed. Moreover, passive smokers are also at risk of developing them [93].

### 5.3. Alcohol Consumption

The relationship between alcohol consumption and the incidence of cardiovascular disease is ambiguous. The amount of alcohol consumption appears to be of key importance in terms of its impact on cardiovascular disease. Excessive alcohol consumption has a devastating effect on health, increasing the possibility of developing hypertension and the risk of death from cardiovascular disease [94,95]. However, studies are showing the benefits of moderate alcohol consumption in the context of cardiovascular disease. Attention is particularly drawn to the consumption of red wine, which, containing both alcohol and polyphenols, reduces oxidative stress and prevents the development of thromboembolic disease [35,96]. It has also been proven that an intake of 25 g of alcohol per week can reduce cardiovascular risk [97].

### 5.4. Stress

Everyday life in the 21st century is filled with stress. In recent years, society has experienced another major stress factor, the development of a pandemic. Chronic exposure to stress has many adverse health effects, mainly on the cardiovascular system. It results in the development of hypertension, rapid progression of atherosclerotic processes, increased insulin resistance, and related obesity [88]. It has been proven that exposure to severe stress increases the risk of death from cardiovascular causes [98]. There is now a strong emphasis on the need for psychological support for people with cardiovascular disease. It is recommended that among patients, in addition to pharmacological and nutritional treatment, psychotherapeutic diagnosis and treatment to improve psychosocial functioning should be carried out [98].

## 6. Conclusions

The latest available literature on the prevention of cardiovascular diseases and their treatment indicates that lifestyle is an extremely important risk factor for the development of cardiovascular diseases, including ischemic heart disease. In the light of the presented studies, it is beneficial for the functioning of the cardiovascular system both as prevention and during the convalescence period:

- eating a well-balanced diet that provides cardioprotective ingredients including polyphenols, sitosterol, vitamin E, CoQ10 s;
- reduce the intake of simple carbohydrates in favor of complex carbohydrates that provide dietary fiber;
- elimination of trans fatty acids and supply of omega-3 polyunsaturated fatty acids with anti-inflammatory effects;
- use of protective dietary models among people at increased cardiovascular risk and patients using both traditional diets such as the DASH diet, the Mediterranean diet, and modern dietary models such as low-carbohydrate diets, the Nordic diet, and the MIND diet;
- engaging in regular physical activity;
- smoking cessation;
- having the ability to manage stress.

The collected publications in the field of lifestyle medicine can be a source of knowledge for nutritionists, physicians, and people involved in physical culture and human mental health to prevent the development of cardiovascular diseases and reduce the risk of death from this cause.

## Figures and Tables

**Table 1:** Selected cardioprotective food components.

CoQ10	Increasing ATP production in the heart muscle cells Strong antioxidant effect Endothelial function improvement Lipid profile improvement	fatty fish, soybeans, spinach, nuts	[19,20]
Omega-3	Lowering the levels of inflammatory markers Improving the blood vessels endothelium functions Reducing the risk of CVD development Lowering triglyceride level Lowering blood pressure Reducting of platelets aggregation	oily sea fish, sea algae, flaxseed, chia seeds	[21,22,23]
Sitosterol	Lipid profile improvement Antioxidant effect	vegetables and fruits, vegetable oils, nuts, legumes	[25]
Vitamin E	Antioxidant effect Reducting of platelets aggregation Reducing the risk of CVD development	vegetables oils, nuts	[27,28]
Polyphenols	Lowering blood pressure Endothelial function improvement Lipid profile improvement Reducing the risk of CVD development	fruits, vegetable oils	[32,33,34]

ATP—Adenosine triphosphate.

**Table 2:** Health benefits of selected dietary models in cardiovascular disease.

Nutrition Model	Health Benefit	Reference
Mediterranean diet	Reducing the risk of cardiovascular disease Reducing the risk of death from cardiovascular disease Improvement of lipid profile Reduction of blood pressure Reducing inflammation	[49,50,51,52,53,54,55,56]
The DASH diet	Reducing the risk of death from cardiovascular disease Reduction of blood pressure Reduction of LDL cholesterol levels Reducing the risk of type 2 diabetes	[48,50,57,58,59]
The MIND Diet	Reduction of oxidative stress Improvement of metabolic functions Prevention of cognitive decline after stroke	[5,48,61,62,64,65]
Vegetarian diets	Reducing the risk of death from cardiovascular disease Reduces the risk of ischemic and hemorrhagic stroke Reduction of LDL cholesterol levels	[67,68,69]
The Nordic diet	Reduction of LDL cholesterol levels Improvement of metabolic functions Sensitization of cells to insulin Reduction of CRP concentration Reduction of blood pressure	[23,73,75]
Low-carbohydrate diets	Weight reduction Reduction of CRP concentration Reduction of triglyceride levels Improving vascular endothelial function Reduction of blood pressure	[76,77,78,79]

LDL—low-density lipoprotein. CRP—C-reactive protein. DASH DIET—Dietary Approaches to Stop Hypertension. MIND diet—Mediterranean–DASH Intervention for Neurodegenerative Delay.

## Data Availability

Not applicable.
